# Functional Outcomes of Thoracolumbar Junction Spine Fractures

**Published:** 2017-05-15

**Authors:** Bradford A. Wall, Alan Moskowitz, M. Camden Whitaker, Teresa L. Jones, Ryan M. Stuckey, Catherine L. Carr-Maben, Alexander CM. Chong

**Affiliations:** 1 Department of Orthopaedics, University of Kansas School of Medicine-Wichita, KS; 2 Department of Orthopaedics, Kansas Orthopaedic Center, PA, Wichita, KS; 3 Department of Orthopaedics, Orthopaedic & Sports Medicine at Cypress, Wichita, KS; 4 Department of Orthopaedics, Via Christi Health Department of Graduate Medical Education, Wichita, KS

**Keywords:** spinal fractures, treatment outcomes, kyphosis, kyphotic curvature

## Abstract

**Introduction:**

Few studies have evaluated the functional outcomes of traumatic thoracic and lumbar vertebral body fractures. This study evaluated the functional and clinical outcomes of patients, who sustained a fracture to the thoracolumbar area of the spine (T10 to L2 region), with ≥ 25° kyphosis versus those with less kyphotic curvature.

**Methods:**

The trauma registry records of two level 1 trauma centers using ICD-9 codes for fracture to the thoracolumbar juncture (T10 to L2 region) were reviewed. Kyphosis angle was measured on the standing lateral thoracolumbar (T1 - L5) radiograph at initial trauma and at clinical follow-up. Functional outcome questionnaires, including the Oswestry Disability Questionnaire (ODQ), the Roland Morris Disability Questionnaire (RMDQ), and the Nottingham Health Profile (NHP), were evaluated at clinical follow-up. Work status and medication used after trauma also were recorded.

**Results:**

A total of 38 patients met the inclusive criteria. Seventeen patients (45%) had ≥ 25° kyphosis and 21 patients (55%) had < 25° kyphosis at follow-up. These two groups were similar based on sex and age. Based on the ODQ Score, the RMDQ Score, and the NHP, no statistically significant differences were detected between the two groups in regards to energy, pain, mobility, emotional reaction, social isolation, and sleep.

**Conclusions:**

Patients who sustained a fracture to the thoracolumbar area of the spine with ≥ 25° kyphosis do not report worse clinical outcomes. When using the kyphosis angle as an indication for surgery, it should be used with caution and not exclusively.

## Introduction

Fractures of thoracic and lumbar spine, especially at the thoracolumbar junction (T10 to L2), often are related to high energy trauma^1^, and represent nearly 90% of traumatic spine fractures.^2^–^5^ The thoracolumbar junction represents a transition zone of the spine, and high energy forces, coupled with the local anatomy, contribute to the high incidence of fractures of this region. Despite the fact that this is a common fracture, the treatment of burst and compression fractures remains controversial regarding the ideal management. Previous studies have proposed treatment guidelines such as canal compromise, neurologic deficit, loss of vertebral body height, and kyphosis as relative indications for operative treatment versus non-operative treatment of this type of injury. The advantages of surgery include better correction of kyphotic deformity, greater initial stability, an opportunity to perform direct or indirect decompression of neural elements, decreased requirements for external immobilization, and an earlier return to work.^6^–^8^ In the body of literature concerning the degree of kyphosis that can be accepted or required, surgical correction continues to be questioned.

To address the questions that surround the treatment of acute thoracolumbar fractures, it is important to elucidate the correlation between residual kyphotic deformity and patient’s functional outcome. Kraemer et al.^4^ performed a retrospective chart review and concluded that patients with kyphosis of greater than 25° were affected more severely and have poorer outcomes. Shen et al.^9^ commented the majority of studies have been on patients with less than 30° kyphosis, therefore, it is impossible to comment on these cases having more severe sagittal angulation in regards to outcome. The purpose of this study was to evaluate the functional and clinical outcomes of patients, who sustained a fracture to the thoracolumbar area of the spine (T10 to L2 region), with greater than or equal to 25° of kyphosis versus those with less kyphotic curvature.

## Methods

The trauma registry records of two Midwest Level 1 regional trauma centers for the last 5.5 years using ICD-9 codes (code: 805.2 – 805.5, 806.20 – 806.40, 806.5, 806.60 – 806.79) were reviewed in a prospective cohort study to identify patients with spinal fracture. Both Level 1 regional trauma centers from which the records were obtained served a rural catchment area for a multi-state region. Before commencing, this study protocol and amendments were reviewed and approved by three local Institutional Review Boards (IRB).

The inclusion criteria for this study were for patients between 18 and 65 years of age with burst or compression vertebral body fracture at the thoracolumbar junction. These fractures resulted from a high energy traumatic event such as fall, motor vehicle accident, motorcycle accident, or sporting event accident. Patients with a fracture that was not located on the vertebral body, had neurovascular involvement, osteoporosis, previous spinal fracture, or prior spinal surgery were excluded from this study.

**Figure 1:**
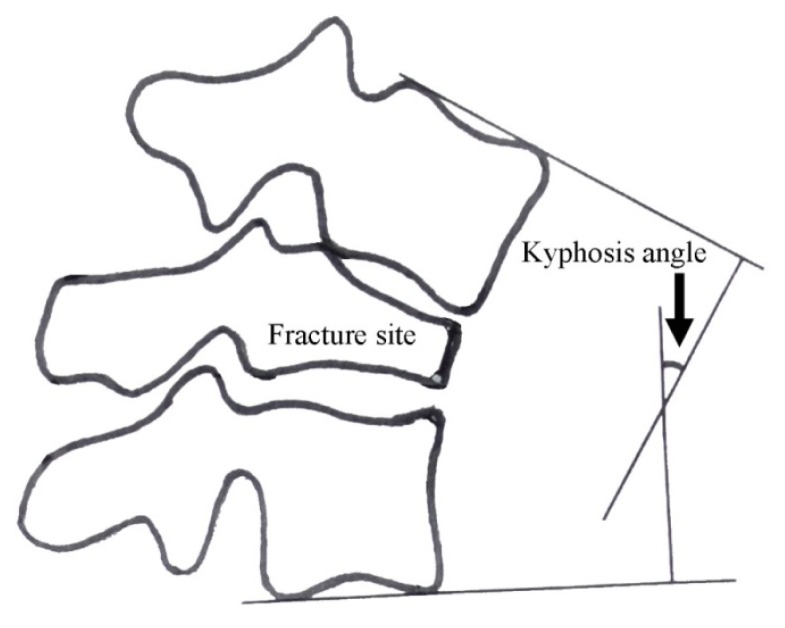
Schematic diagram of kyphosis angle measurement on lateral thoracolumbar radiograph.

The standing lateral thoracolumbar (T1 - L5) radiograph of potential patients was reviewed (at initial trauma), and was used to measure the amount of kyphosis at the fracture site from the next adjacent intact vertebrae above and below using the Cobb method (Figure 1). This measuring method is similar to one previously reported.^10^ Each potential patient was contacted through a recruitment letter or by telephone, and reimbursement for their research-related expenses was offered to recruit participants.

A clinical follow-up evaluation (at least four months post-trauma) was performed using standing lateral thoracolumbar radiographs to measure the post-trauma kyphosis angle and functional outcome questionnaires to determine level of disability and general health status. These functional outcome questionnaires included the Oswestry Disability Questionnaire (ODQ), the Roland Morris Disability Questionnaire (RMDQ), and the Nottingham Health Profile (NHP). Work status and medication use after the trauma also was collected. The ODQ is a time-tested outcome assessment tool that is used to measure a patient’s impairment and quality of life. The RMDQ is a self-administered disability measure in which greater levels of disability are reflected by higher numbers on a 24-point scale. The RMDQ yields reliable measurements, which are valid for inferring the level of disability, and sensitive to change over time for groups of patients with low back pain. The NHP is a general patient-reported outcome measure which seeks to measure subjective health status and is a questionnaire designed to measure a patient’s view of their own health status in a number of areas in regards to energy, pain, physical mobility, emotional reaction, social isolation, and sleep. These questionnaires are considered the “gold standard” of low back functional outcome measuring tools.^11^–^13^

## Statistical Analysis

Statistical evaluation included the use of the non-parametric Mann-Whitney U statistic using SPSS software (Version 19.0; SPSS Inc., Chicago, IL) to compare those with greater kyphotic measurements versus those with lesser kyphotic measurements. The Chi-square statistic also was used to determine if a distribution of observed frequencies differed from theoretical expected frequencies where the dependent and independent variables were nominal or ordinal measures. The level of significant difference was defined as p < 0.05.

## Results

A total of 38 patients meeting criteria was comprised of 21 men (55%) and 17 women (45%). Seventeen (45%) of the 38 patients were those with ≥ 25° kyphosis at follow-up, with five of those patients (29%) presenting initially and 12 patients (71%) progressing to an increase in kyphotic measurement at follow-up. There were nine male (53%) and eight female (47%) in this subgroup with mean age of 37 ± 15 years old (range: 18 – 63 years old). Seven patients (41%) of the 17 had a record of open reduction internal fixation (ORIF) surgery at the time of acute hospitalization, with the remainder being treated with conservative therapies prior to hospital dismissal (Table 1).

Twenty-one (55%) of the 38 patients were those with < 25° kyphosis at follow-up, whereas only one of these patients (5%) presented initially with ≥ 25° kyphosis. There were 12 males (57%) and nine females (43%) in this subgroup with mean age of 40 ± 16 years old (range: 18 – 64 years old). Five patients (24%) had a surgery at the time of acute hospitalization (two patients had ORIF and three had kyphoplasty or vertebraplasty) with the remainder 16 patients (76%) selected with conservative therapies prior to hospital dismissal. Table 1 shows a complete demographic summary and descriptive statistics.

## Oswestry Disability Questionnaire (ODQ) Score

**Figure 2:**
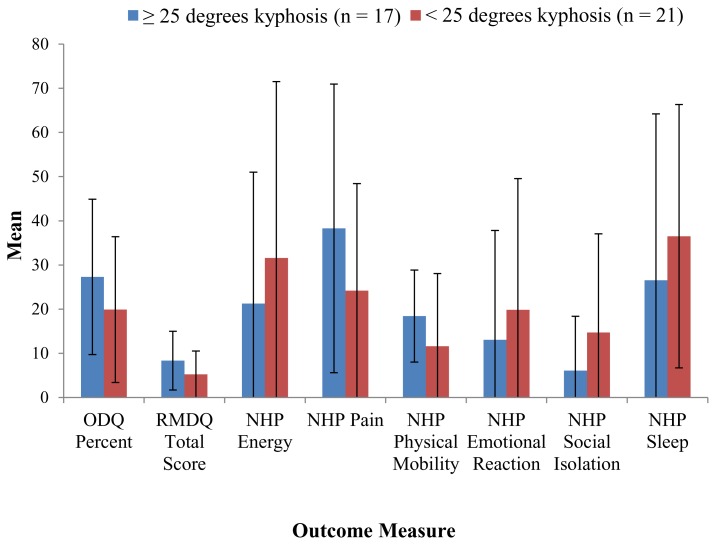
Mean outcome measures stratified by binary follow-up angle measurement with standard deviation.

The overall ODQ score was calculated as a percent according to standardized methods. The overall mean percent score for the group of 38 patients, who sustained a fracture to the thoracolumbar area of the spine (T10 to L2 region), was 23% ± 17. When stratified by degrees of kyphosis, the ≥ 25° kyphosis group was higher at 27% ± 18 as compared to the < 25° kyphosis group at 20% ± 17. However, no statistically significant difference was detected (p = 0.17, Figure 2).

## Roland and Morris Disability Questionnaire (RMDQ) Score

The RMDQ score was summed according to standardized methods. The average score was 6.7 ± 6.1. All strata were compared for association with none showing a significant difference in terms of disability (Figure 2). There was a trend, however, in operative patients with < 25° kyphosis group having a significant increase in disability when compared to the same degree of kyphosis non-operative patients.

## Nottingham Health Profile (NHP) Score

The NHP score was calculated for the six major domains according to standardized methods, which included weighted scoring. For the overall, the six domains yielded a mean and standard deviation as follows: NHP Energy = 25.7 ± 34.2, NHP Pain = 30.6 ± 28.9, NHP Physical Mobility = 14.9 ± 14.1, NHP Emotional Reaction = 15.8 ± 26.6, NHP Social Isolation = 10.1 ± 18.2, and NHP Sleep = 32.8 ± 34.0. Chi-square statistic testing showed no statistically significant differences except for the NHP Physical Mobility which approached significance (p = 0.05; Figure 2).

## Work Status

At final follow-up, 23 patients (61%) of the 38 patients reported returning to their full-time work status, with another six patients (16%) listing part-time employment. Of those patients with ≥ 25° kyphosis at follow-up, one patient (6%) was unable to work due to back pain, and two patients (12%) reported not returning by choice. There was no patient with < 25° kyphosis at follow-up that reported being unable to work after the trauma (Table 2). No significant difference, however, was detected between these two groups.

## Medication Used after the Trauma

Of those reporting medication use at follow-up, 17 patients used at least one narcotic for pain (12 patients used hydrocodone/acetaminophen; four patients used oxycodone/acetaminophen; and one patient used codeine/acetaminophen). Two of the 17 patients reported use of different combination types of narcotics: hydrocodone/acetaminophen and oxycodone/acetaminophen in combination and oxycodone/acetaminophen and codeine/acetaminophen in combination. None reported using more than two narcotic drugs in combination.

Nine of the 17 patients reporting narcotic use used anti-inflammatory medications, with one patient taking additional acetaminophen, two patients taking aspirin, five patients taking ibuprofen, and one patient taking celecoxib. There were an additional 11 patients that took only an anti-inflammatory with one patient taking naproxen, four patients taking aspirin, five patients taking ibuprofen, and one patient taking tramadol. Of those only taking an anti-inflammatory, three patients also took a second anti-inflammatory, ibuprofen.

Of those reporting medication use, 10 patients reported taking an antidepressant at the time of the two-year mean follow-up. One patient was taking fluoxetine alone (no other medication), three patients reported taking venlafaxine hydrochloride extended-release along with an anti-inflammatory (alprazolam, ibuprofen, or clorazepate), and six patients reported taking one of five antidepressants along with a narcotic medication (one patient taking quetiapine, one patient taking paxil, two taking fluoxetine, one taking duloxetine, and one taking sertroline). Of the eight reporting use of muscle relaxants, all were in combination with narcotic medications, five with cyclobenzaprine, one with diazepam, one with valium, and one with metaxalone.

## Discussion

The decision to treat acute thoracic and lumbar spine fractures, especially at the thoracolumbar junction (T10 to L2), operatively or non-operatively based on kyphotic deformity of the patient, remains controversial. Conservative treatment is usually the method of choice as it was related to lower costs and lower complication rates.^4^,^14^–^27^ This type of treatment for unstable fractures, however, is associated with high risk of neurologic deterioration, putting neural elements at risk of injury, and potential development of progressive instability.^14^,^16^,^19^,^26^–^31^ Operative stabilization of the spine is preferred in those patients who need correction of the kyphotic deformity, thereby reducing mechanical back pain and allowing early patient mobilization.^1^,^6^,^32^–^36^

Kyphotic deformity at the thoracolumbar junction has been a more controversial matter as there have been conflicting studies as to the amount of kyphosis leading to poor outcomes and necessitating operative treatment. Gertzbein et al.^37^ reported a positive relationship between kyphotic deformities of 30° or more and back pain at both 1- and 2-year follow-up of thoracic and lumbar fractures. In their study, they concluded kyphotic deformity of greater than 30° was associated with an increased incidence of more intense back pain; however, this study did not subdivide the type of fractures. Krompinger et al.^18^ stated that if the kyphosis angle was less than 30° and spinal canal narrowing was less than 50% then these could be defined as stable. They reported that 36% of thoracolumbar burst fractures progressed 10° or more at follow-up; however, the remaining residual deformity was not correlated with symptoms at follow-up. Reid et al.^22^ concluded that it was necessary to treat patients operatively with burst fractures if these patients have neurologic deficits or a kyphosis angle more than 35°. Shen et al.^4^,^29^ concluded there was a poor correlation between clinical results and kyphosis greater than 30°, and Cantor et al.^15^ stated that fractures without neurologic deficit, with kyphosis less than 30° and height loss less than 50%, were defined as stable. The findings of the present study concur with these previous studies that there was no association between the kyphotic deformity ≥ 25° and functional and clinical outcomes of patients.

Several questions and limitations can be raised concerning the outcome of this study. This study was a prospective cohort study, but not randomized. With follow-up period of 2.3 years, the results must be considered short-term outcomes. One other weakness of present study was that a low percentage of the trauma patient population (38 patients) participated, and there were a relatively small number of patients with ≥ 25° of kyphosis deformity. Nevertheless, the numbers of these patients exceed those that have been reported in prior reports. This study also was limited in that there was no standardized conservative treatment that was strictly practiced, thus treatment options could be another possible factor affecting the clinical outcomes.

## Conclusions

The functional and clinical outcomes of patients who sustained a fracture to the thoracolumbar area of the spine (T10 to L2 region) with ≥ 25° of kyphosis were not considerably different from that of those with < 25° of kyphosis. Based on the results of this study, patients who sustained a fracture to the thoracolumbar area of the spine with ≥ 25° of kyphosis do not appear to report worse clinical outcomes. It is advised, however, that when using this criterion as a sole indication for surgery, it should be used with caution and not exclusively. Further investigation of this patient population with functional outcome measures is required to support the conclusion of this study.

## Figures and Tables

**Table 1:** Demographic summary and descriptive statistics.

Thoracolumbar Juncture Fracture T10 to L2 (n = 38)		Follow-up angle ≥ 25 degrees (n = 17)	Follow-up angle< 25 degrees (n = 21)	p-value
Initial Angle ≥ 25 degrees -- Yes / No		5 (29%) / 12 (71%)	1 (5%) / 20 (95%)	0.04*^S^
Initial Angle (degrees)		21.1 ± 9.4 (range: 7 to 45)	8.0 ± 9.4 (range: −7 to 26)	0.00‡^S^
Mean Follow-Up Angle (degrees)		34.4 ± 7.8 (range: 25 to 47)	10.4 ± 8.3 (range: −5 to 24)	0.00‡^S^
Gender -- Male / Female		9 (53%) / 8 (47%)	12 (57%) / 9 (43%)	0.80*^NS^
Age at Injury (years)		37.3 ± 15.3 (range: 18 to 63)	40.5 ± 15.7 (range: 18 to 64)	0.47‡^NS^
Surgical Treatment Type	Kyphoplasty/Vertebraplasty	0 (0%)	3 (14%)	0.03*^S^
	ORIF	7 (41%)	2 (10%)	
	No treatment	10 (59%)	16 (76%)	

* Significance testing Chi-square statistic (*NS = not significant/*S = significant, p < 0.05)

‡ Significance testing Mann-Whitney U statistic (‡NS = not significant/‡S = significant p < 0.05)

**Table 2:** Work status summary.

Thoracolumbar Juncture Fracture T10 to L2 (n = 38)	Follow-up angle ≥ 25 degrees (n= 17)	Follow-up angle < 25 degrees (n = 21)	p-value
Work Full-time	8 (47%)	15 (71%)	0.37*^NS^
Work Part-time	4 (24%)	2 (10%)	
Seeking Employment	1 (6%)	1 (5%)	
Not working by choice	2 (12%)	3 (14%)	
Unable to work due to back problem	1 (6%)	0 (0%)	
Unable to work NOT due to back problem	1 (6%)	0 (0%)	

* Chi-square statistic (*NS = not significant/*S=significant p < 0.05)
